# Genetic polymorphisms in *TERT* are associated with increased risk of esophageal cancer

**DOI:** 10.18632/oncotarget.14451

**Published:** 2017-01-02

**Authors:** Yifei Wu, Mengdan Yan, Jing Li, Jingjie Li, Zhengshuai Chen, Peng Chen, Bin Li, Fulin Chen, Tianbo Jin, Chao Chen

**Affiliations:** ^1^ Key Laboratory of Resource Biology and Biotechnology in Western China (Northwest University), Ministry of Education, Xi’an, Shaanxi 710069, China; ^2^ Xi’an Tiangen Precision Medical Institute, Xi’an, Shaanxi 710075, China; ^3^ Institution of Basic Medical Science, Xi'an Medical University, Xi’an, Shaanxi 710021, China

**Keywords:** TERT, SNP, esophageal cancer, susceptibility, northwest Chinese population

## Abstract

Single nucleotide polymorphisms (SNPs) in *TERT* may be associated with susceptibility to esophageal cancer. In this study, we analyzed the association between *TERT* SNPs and risk of esophageal cancer in 386 esophageal cancer patients and 495 healthy subjects from the Xi’an area of China. Of the four SNPs examined, rs10069690 and rs2242652 were correlated with esophageal cancer risk. Additionally, after adjusting for age and gender, the “T_rs10069690_A_rs2242652_”, “T_rs10069690_G_rs2242652_” haplotypes were associated with an increased risk of esophageal cancer, while the and “C_rs10069690_G_rs2242652_” haplotype was associated with a decreased risk of esophageal cancer. These findings suggest that TERT polymorphisms may contribute to the development of esophageal cancer.

## INTRODUCTION

Esophageal cancer is the sixth leading cause of cancer-related death worldwide, and an estimated 500,000 new cases are diagnosed each year [1]. In China, esophageal cancer was the fifth most commonly diagnosed cancer and the fourth leading cause of cancer-related death in 2009 [2]. Esophageal cancer is very aggressive and is associated with a poor prognosis [3]. Esophageal cancer can be categorized as one of two histological types, adenocarcinoma and squamous cell carcinoma (SCC), each of which have distinct etiologies and specific risk factors [4]. Esophageal squamous cell carcinoma (ESCC) accounts for more than 90 percent of esophageal cancers [5] and is the major histological subtype in East Asian countries [6]. Smoking and alcohol consumption are major risk factors for ESCC [7]. The incidence of esophageal cancer has increased markedly worldwide in recent decades, and this increase can be mainly attributed to higher incidences of esophageal adenocarcinoma in North America [8, 9], Europe [10], and Japan [11]. Meanwhile, the incidence of ESCC has remained stable or declined [8, 10].

Telomerase, a reverse transcriptase that carries its own templates, maintains telomere length by synthesizing telomeric DNA repeats. Many events can result in telomere dysfunctions, including gradual shortening caused by incomplete chromosome replication, oxidative DNA damage, or mutations in structural proteins, such as *TERT* [12]. *TERT* is the reverse transcriptase catalytic subunit of telomerase, and the activation of both *TERT* and telomerase plays a key role in cellular immortalization and transformation [13]. In addition, multiple independent variants at the *TERT* locus are associated with telomere length and risk of malignant tumors [14]. *TERT* uses the RNA subunit of telomerase as a template for the synthesis of single-stranded DNA in the telomeric region of the chromosome, resulting in tandem nucleotide repeats that prevent shortening of the chromosome. Telomerase activity, which is elevated in most cancer cells, may block apoptosis or senescence in cancer cells [15]. Several studies have shown that TERT promotes tumor invasion and metastasis in gastric, liver, and esophageal cancer [16–18].

In this study, we conducted an extensive association analysis to evaluate the roles of *TERT* gene polymorphisms and haplotypes on susceptibility to esophageal cancer in a population of northwestern Chinese patients from a single case-control study. Four *TERT* single nucleotide polymorphisms (SNPs) were examined in this association analysis in an attempt to identify markers that might guide intervention decisions and improve patient survival.

## RESULTS

The genotypes of 881 participants, including 386 esophageal cancer patients and 495 controls, were analyzed in this study. Basic patient characteristics (gender and age) are listed in Table 1. 79.8% of the esophageal cancer patients were men and 20.2% were women, while 36.4% of the controls were men and 63.6% were women. The mean ages were 60.68 (±8.954) years for esophageal cancer patients and 54.48 (±9.438) years for controls.

**Table 1:** General characteristics the of this study population

Variable	Cases	Controls
	n = 386	n = 495
**Gender**		
Male	308 (79.8%)	180 (36.4%)
Female	78 (20.2%)	315 (63.6%)
**Age**, year (mean ±SD)	60.68 ±8.954	54.48 ±9.438

Four SNPs in the *TERT* gene were genotyped in the esophageal cancer patients and the healthy controls. Table 2 summarizes the basic characteristics of these *TERT* SNPs in the study population. All four SNPs conformed to HWE in the control group (*p* > 0.05), and two-sided Pearson chi-square tests were used to identify differences in allele frequency distributions between esophageal cancer patients and controls. The rs10069690, rs2242652, and rs2853676 SNPs were associated with 1.70-fold (95% CI, 1.33 - 12.18, *p* = 2.50E-05), 1.48-fold (95% CI, 1.17 - 1.89, *p* = 0.001), and 1.33-fold (95% CI, 1.04 – 1.70, P = 0.023) increases in the risk of developing esophageal cancer, respectively. Two of these SNPs (rs10069690 and rs2242652) were still correlated with esophageal cancer in the allelic model after Bonferroni correction.

**Table 2:** Frequency distributions of *TERT* alleles and their associations with esophageal cancer risk

SNP ID	Position	Allele A*/B	MAF		HWE-*p*	ORs (95% CI)	^a^*p*-value	^b^*p-*value
			Cases	Controls				
rs10069690	1279790	T/C	0.22	0.14	0.27	1.70 (1.33 - 2.18)	**2.50E-05**	**1.0E-04**
rs2242652	1280028	A/G	0.23	0.16	1	1.48 (1.17 - 1.89)	**0.001**	**0.004**
rs2853677	1287194	G/A	0.40	0.38	0.44	1.14 (0.93 - 1.38)	0.200	0.800
rs2853676	1288547	T/C	0.20	0.16	0.50	1.33 (1.04 - 1.70)	**0.023**	0.092

**Abbreviations:** SNP = single nucleotide polymorphism, CI = confidence interval, HWE = Hardy-Weinberg Equilibrium, MAF = minor allele frequency, ORs = odds ratios.

**Notes:** *Minor allele; ^a^*p*-values were calculated from two-sided Pearson chi-square tests for either allele frequency and ^b^*p*-values were adjusted by Bonferroni correction; *p*-value < 0.05 indicates statistical significance.

Next, we examined the association between *TERT* SNPs and esophageal cancer risk using genetic models (Table 3); *p*-values were calculated using the Wald test. The “T/C-T/T” genotype at the rs10069690 SNP increased the risk of esophageal cancer in the dominant model (OR = 1.95; 95% CI, 1.43 – 2.73, *p* = 5.89E-06), and the “T” allele increased the risk of esophageal in the log-additive model (OR = 1.71; 95% CI, 1.33 – 2.20, *p* = 3.02E-05). These relationships in both the dominant model (*p* = 4.33E-05) and the log-additive model (*p* < 0.001) remained significant after adjustments for age and gender.

**Table 3:** Genotypic model analysis of relationship between SNPs and esophageal cancer risk

SNP	Model	Genotype	Cases	Controls	Without adjustment		With adjustment		^b^*p*-value
					ORs (95% CI)	*p-value*	ORs (95% CI)	^a^*p*-value	
rs10069690	Dominant	C/C	227	359	1				
		T/C-T/T	155	126	1.95 (1.46 - 2.60)	**5.89E-06**	1.97 (1.43- 2.73)	**4.33E-05**	**1.732E-04**
	Recessive	C/C-C/T	368	472	1				
		TT	14	13	1.39 (0.64 - 2.96)	0.41	1.48 (0.62 - 3.51)	0.3794	1
	Log-additive	-	-	-	1.71 (1.33 - 2.20)	**3.02E-05**	1.73 (1.31 - 2.31)	**< 0.001**	**<0.005**
rs2242652	Dominant	G/G	224	346	1				
		G/A-A/A	158	149	1.64 (1.24 - 2.17)	**5.5E-04**	1.59 (1.16 - 2.18)	**0.004**	**0.016**
	Recessive	G/G-G/A	368	482	1				
		A/A	14	13	1.41 (0.66 - 3.04)	0.38	1.48 (0.62 - 3.50)	0.378	1
	Log-additive	-	-	-	1.51 (1.18 - 1.93)	**0.001**	1.48 (1.12 - 1.95)	**0.006**	**0.024**
rs2853676	Dominant	C/C	248	355	1				
		C/T-T/T	138	140	1.41 (1.06 - 1.88)	0.18	1.44 (1.05 - 1.99)	0.026	0.104
	Recessive	C/T-T/T	372	481	1				
		T/T	14	14	1.29 (0.61 - 2.75)	0.5	1.33 (0.56 - 3.12)	0.520	1
	Log-additive	-	-	-	1.33 (1.04 - 1.70)	0.24	1.36 (1.02 - 1.79)	0.034	1
rs2853677	Dominant	AA	140	202	1		1		
		A/G-G/G	241	293	1.19 (0.90 -1.56)	0.22	1.26 (0.92 – 1.71)	0.15	0.60
	Recessive	A/G-A/A	319	424	1		1		
		G/G	62	71	1.16 (0.80 - 1.68)	0.43	1.39 (0.91 - 2.12)	0.13	0.52
	Log-additive	-	-	-	1.13 (0.93 - 1.37)	0.21	1.22 (0.98 – 1.52)	0.07	0.28

**Abbreviations:** CI = confidence interval, OR = odds ratio, SNP = single nucleotide polymorphism.

**Notes:** *p-*values were calculated by *Wald* test and *p-*value < 0.05 indicates statistical significance;

^a^*p*-value were adjusted by gender and age and ^b^*p*-value were adjusted by Bonferroni correction.

For the rs2242652 SNP, the “G/A-A/A” genotype increased the risk of esophageal cancer 1.64-fold (95% CI, 1.24 - 2.197; *p* = 5.5E-04) in the dominant model, and the “A” allele increased the risk 1.48-fold (95% CI, 1.18 - 1.93; *p* = 0.001) in the log-additive model. In addition, after adjustment for age and gender, the “G/A-A/A” genotype increased the risk of lung cancer 1.59-fold (*p* = 0.004) and the “A” allele increased the risk 1.48-fold (*p* = 0.006).

Interestingly, a third SNP (rs2853676) was associated with susceptibility to esophageal cancer after adjustment for age and gender. Specifically, the “C/T-T/T” genotype increased the risk of esophageal cancer in the dominant model (OR = 1.44; 95% CI, 1.05 – 1.99, *p* = 0.026), and the “T” allele increased the risk in the log-additive model (OR = 1.36; 95% CI, 1.02 – 1.79, *p* = 0.034). After the Bonferroni correction, mutations at rs10069690 (dominant *p* = 1.732E-04; additive *p* < 0.005) and rs2242652 (dominant *p* = 0.016; additive *p* = 0.024) were still associated with an increased risk of esophageal cancer.

**Figure 1:**
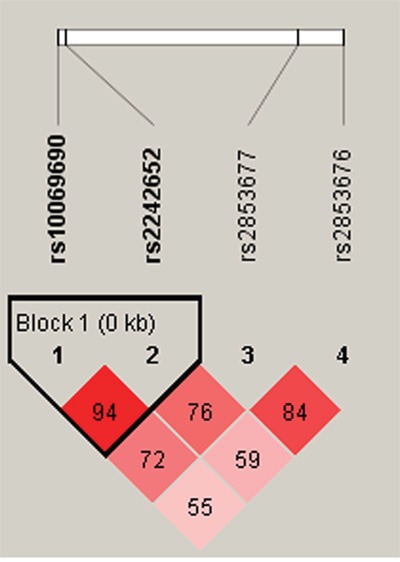
Linkage disequilibrium patterns for four *TERT SNPs*

*TERT* polymorphisms were further characterized using linkage disequilibrium (LD) and haplotype analyses. LD was determined pairwise among all four SNPs, the haplotype structure of the *TERT* gene was analyzed, and single LD blocks consisting of two SNPs (rs10069690 and 2242652) were detected (Figure 1).

Finally, a haplotype-based association study was performed to examine associations between *TERT* haplotype and risk of esophageal cancer (Table 4), and *p*-values were calculated using the Wald test. After adjusting for age and gender, the “TA” (95% CI, 1.91 – 2.12; *p* = 0.002) and “TG” (95% CI, 1.20 – 11.1; *p* = 0.022) haplotypes increased the risk of esophageal cancer 1.59-fold and 3.66-fold, respectively. In contrast, the “CG” haplotype was associated with a 0.61-fold decrease in the risk of esophageal cancer (95% CI, 0.46 – 0.81; *p* < 0.001).

**Table 4:** Haplotype analysis results in this study

Haplotype		Frequency		Without adjustment		With adjustment	
rs10069690	rs2242652	Cases	Controls	ORs (95% CI)	*p*-value	ORs (95% CI)	*p*-value*
T	A	0.206	0.137	1.63 (1.26 - 2.11)	**< 0.001**	1.59 (1.91 - 2.12)	**0.002**
C	A	0.017	0.02	0.87 (0.43 - 1.79)	0.71	0.83 (0.37 - 1.84)	0.641
T	G	0.015	0.006	2.38 (0.87 - 6.51)	0.08	3.66 (1.20 - 11.1)	**0.022**
C	G	0.762	0.837	0.61 (0.47 - 0.78)	**< 0.001**	0.61 (0.46 - 0.81)	**< 0.001**

**Abbreviations:** CI = confidence interval, OR = odds ratio, SNP = single nucleotide polymorphism.

**Notes:** *p*-values were calculated by *Wald* test and *p*-value < 0.05 indicates statistical significance;

*Adjusted by gender and age.

## DISCUSSION

In the present study, we systematically examined the impact of four SNPs in the *TERT* loci on susceptibility to esophageal cancer. We found that the rs10069690, rs2242652, and rs2853676 *TERT* genetic polymorphisms were associated with an increased risk of esophageal cancer in a northwestern Chinese patient population. To the best of our knowledge, this is the first case-control study to investigate this association.

The incidence of esophageal cancer is increasing worldwide. For example, in the United States, the incidence of esophageal cancer has increased six-fold over the last three decades [19]. Meanwhile, the incidence of SCC has remained stable in Europe and the United States [8, 10]. Furthermore, while the incidence of SCC remained constant in males, it increased slightly in females, in whom rates of smoking have also increased in recent decades. Genetic and environmental factors both play important roles in cancer pathogenesis. For example, nitrosamine exposure resulting from tobacco use has been associated with an increased risk of esophageal cancer [20, 21]. In addition, alcohol consumption is a risk factor for ESCC, but not for adenocarcinoma [21, 22]. Other important factors include low socioeconomic status, poor oral hygiene, and nutritional deficiencies [23–25].

As the main catalytic subunit of telomerase, *TERT* is essential for the maintenance of telomere DNA length in chromosomes [13]. Telomerase is an RNA-dependent DNA polymerase that synthesizes repeating TTAGGG DNA sequences, which bind many specialized proteins to the ends of the chromosome [26]. Telomeres prevent coding sequence erosion and protect chromosomes from rearrangements, fusion, and genome instability by aiding in complete chromosome replication and regulating gene expression [27]. The activation of telomerase is a vital step during cellular immortalization and malignant transformation in human cells, and many human malignancies are characterized by elevated TERT expression. TERT may therefore play an important role in cancer pathogenesis [13, 28]. The TERT gene sequence in general is thought to be indicative of an individual’s susceptibility to cancer [29–31], and epidemiological studies have identified associations between specific TERT polymorphisms and cancer development [29, 30].

Rs10069690 was originally discovered in a genome-wide association study of AAs [32], and we suspected that this SNP might also affect the risk of developing esophageal cancer. Another previous study found that the variant allele at *TERT* rs4246742 was inversely associated with breast cancer risk, while positive associations were found for rs10069690 (OR = 1.13), rs2242652 (OR = 1.51), and rs2853676 (OR 1.23) [33]. The rs2853676 polymorphism is also associated with an increased risk of glioma [34]. The additive ORs of the rs2853676 and rs2242652 SNPs for the risk of melanoma were 1.43 and 1.50, respectively [35]. Certain rs10069690 and rs2242652 SNP alleles are also associated with increased risk of estrogen receptor-negative cancers [14, 36]. Furthermore, recent studies have shown that rs2853677 is associated with the risk of lung adenocarcinoma in Japanese [37] and European [38] populations. Here, we found that the rs10069690, rs2242652, and rs2853676 *TERT* genetic polymorphisms were associated with an increased risk of esophageal cancer in a northwestern Chinese patient population; subsequent studies should be conducted to examine these associations in patients from other regions and ethnic groups.

We also identified an LD block consisting of the rs10069690 and 2242652 SNPs, both of which increased susceptibility to esophageal cancer. A previous study found that the rare G_rs4246742_A_rs2736100_T_rs2853676_ *TERT* haplotype was directly associated with telomere length [39]. Future studies should evaluate the association between the rs4246742 andrs2736100 SNPs and susceptibility to esophageal cancer.

Some limitations of the present study should be considered when interpreting the results. First, although there was sufficient statistical power for our analyses, the sample size in this study was relatively small. Second, associations between *TERT* polymorphisms and clinicopathological disease type were not evaluated in this study, and larger, well-designed studies are needed to confirm the associations observed here. Finally, both esophageal cancer patients and controls were enrolled at a single hospital and therefore may not be representative of the general population. Additional studies are needed to clarify the genetic mechanisms underlying esophageal carcinogenesis by fine-mapping the susceptibility regions of the variants.

In conclusion, we demonstrated that the rs10069690, rs2242652, and rs2853676 genetic polymorphisms in the *TERT* loci are associated with an increased risk of esophageal cancer in a northwestern Chinese patient population. Our results demonstrate the complex genetic regulation of telomere biology and the crucial role of telomerase in carcinogenesis. These results may also help improve the understanding of inter-population differences in esophageal cancer etiology, although the biological functions of these TERT SNPs need to be investigated in future studies. Finally, the esophageal cancer-associated molecular markers identified here might be useful as diagnostic and prognostic markers for esophageal cancer patient in future clinical studies.

## MATERIALS AND METHODS

### Ethics and consent

All subjects were informed of the purpose of the study and the experimental procedures involved. The Human Research Committee of the Tangdu Hospital for Approval of Research Involving Human Subjects approved the use of human tissue in this study. We also obtained signed informed consent from all participants.

### Study participants

386 patients were recruited between August 2012 and July 2016 to participate in an ongoing molecular epidemiological study at the Department of Respiratory Physicians of the Tangdu Hospital affiliated with The Fourth Military Medical University in Xi’an, China. All subjects were screened by examining endoscopic staining with 1.2% iodine solution, and biopsies were taken from non-staining stained mucosal areas for each patient. Tissue sections were reviewed by two experienced pathologists to ensure that tumor cell purity was greater than 50% and to confirm histological type. None of the patients had a history of other cancers, nor did they receive chemotherapy or radiotherapy prior to this study. Patients with any comorbidity, such as diabetes mellitus, hypertension, or an endocrine disorder, were excluded. We also recruited 495 unrelated healthy individuals at random during the same period at the medical center of Tangdu Hospital to serve as controls. All study participants lived in or near Xi’an. Control group individuals had no history of known medical illness or hereditary disorders and were not taking any medications.

### SNP selection and genotyping

We examined four single nucleotide polymorphisms (SNPs) in *TERT* that had minor allele frequencies (MAF) greater than 5%. Samples were centrifuged and stored at -80° until analysis. We extracted genomic DNA from peripheral blood samples using the GoldMag-Mini Whole Blood Genomic DNA Purification Kit (GoldMag Ltd. Xi'an, China) according to the manufacturer's protocol and measured DNA concentrations using a NanoDrop 2000. Sequenom MassARRAY Assay Design 3.0 Software was used to design primers for amplification and extension reactions [40]. SNP genotyping was performed using a Sequenom MassARRAY RS1000 according to the manufacturer’s standard protocol [40]. Finally, Sequenom Typer 4.0 Software was used for data management and analysis [40, 41].

### Statistical analysis

Departure from Hardy-Weinberg Equilibrium (HWE) was assessed for the frequency of each SNP using an exact test on the control subjects. Differences in genotype frequencies between the esophageal cancer and control groups were evaluated using the Chi-square test [42]. Microsoft Excel and the SPSS 17.0 statistical package (SPSS, Chicago, IL) were used for statistical analyses. All *p*-values presented in this study are two-sided; *p* ≤ 0.05 was considered statistically significant. Odds ratios (OR) and 95% confidence intervals (CI) were calculated using unconditional logistic regression analyses [43]. The web-based software SNP Stats was used to identify associations between SNPs and the risk of esophageal cancer in three genetic models (dominant, recessive, and additive) [44]. We used the Haploview software package (version 4.2) and the SHEsis software platform (http://www.nhgg.org/analysis/) to analyze linkage disequilibrium, haplotype construction, and genetic associations at polymorphism loci [45, 46].
